# Medicare-Covered Services Near the End of Life in Medicare Advantage vs Traditional Medicare

**DOI:** 10.1001/jamahealthforum.2024.1777

**Published:** 2024-07-19

**Authors:** Lauren Hersch Nicholas, Stacy M. Fischer, Alicia I. Arbaje, Marcelo Coca Perraillon, Christine D. Jones, Daniel Polsky

**Affiliations:** 1 University of Colorado Medical School, Aurora; 2 Johns Hopkins School of Medicine, Johns Hopkins Bloomberg School of Public, Baltimore, Maryland; 3 Colorado School of Public Health, Aurora; 4 Division of Hospital Medicine and Geriatric Medicine, Department of Medicine, University of Colorado School of Medicine, Aurora; 5 Rocky Mountain Regional VA Medical Center, Aurora, Colorado; 6 Johns Hopkins Bloomberg School of Public Health, Baltimore, Maryland

## Abstract

**Importance:**

Financial incentives in Medicare Advantage (MA), the managed care alternative to traditional Medicare (TM), were designed to reduce overutilization. For patients near the end of life (EOL), MA incentives may reduce potentially burdensome care and encourage hospice but could also restrict access to costly but necessary services.

**Objective:**

To compare receipt of potentially burdensome treatments and transfers and potentially necessary postacute services in the last 6 months of life in individuals with MA vs TM.

**Design, Setting, and Participants:**

A retrospective analysis of Medicare claims data among older Medicare beneficiaries who died between 2016 and 2018. The study included Medicare decedents aged 66 years or older covered by TM (n = 659 135) or MA (n = 360 430). All decedents and the subset of decedents with 1 or more emergent hospitalizations with a life-limiting condition (cancer, dementia, end-stage organ failure) that would likely qualify for hospice care were included.

**Exposure:**

MA enrollment.

**Main Outcomes:**

Receipt of potentially burdensome hospitalizations and treatments; receipt of postdischarge home and facility care.

**Results:**

The study included 659 135 TM enrollees (mean [SD] age at death, 83.3 [9.0] years, 54% female, 15.1% non-White, 55% with 1 or more life-limiting condition) and 360 430 MA enrollees (mean [SD] age at death 82.5 [8.7] years, 53% female, 19.3% non-White, 49% with 1 or more life-limiting condition). After regression adjustment, MA enrollees were less likely to receive potentially burdensome treatments (−1.6 percentage points (pp); 95% CI, −2.1 to −1.1) and less likely to die in a hospital (−3.3 pp; 95% CI, −4.0 to −2.7) compared with TM. However, when hospitalized, MA enrollees were more likely to die in the hospital (adjusted difference, 1.3 pp; 95% CI, 1.1-1.5) and less likely to be transferred to rehabilitative or skilled nursing facilities (−5.2 pp; 95% CI, −5.7 to −4.6). Higher rates of home health and home hospice among those discharged home offset half of the decline in facility use. Results were unchanged in the life-limiting conditions sample.

**Conclusions:**

MA enrollment was associated with lower rates of potentially burdensome and facility-based care near the EOL. Greater use of home-based care may improve quality of care but may also leave patients without adequate assistance after hospitalization.

## Key Points

**Question:**

Does end-of-life (EOL) care differ for Medicare beneficiaries in Medicare Advantage (MA) vs traditional Medicare (TM)?

**Findings:**

In this population-based, cross-sectional study of 360 430 MA decedents and 659 135 TM decedents, MA decedents were less likely to receive potentially burdensome treatments or transfers in the last 6 months of life, and more likely to receive hospice care; if hospitalized in the last 6 months of life, MA decedents were more likely to die in the hospital, and less likely to receive facility-based care postdischarge. Higher rates of home care among those discharged home with MA offset less than half of the lower rate of discharge to skilled nursing facilities.

**Meaning:**

Receipt of less facility-based and potentially burdensome care near EOL may improve quality of care for MA decedents; however, the greater reliance on home-based care may leave patients with unmet needs or relying on informal care assistance.

## Teaser

This cross-sectional study compares potentially burdensome hospital treatments and hospitalizations and postdischarge care for Medicare decedents in Medicare advantage vs traditional Medicare at the end of life.

## Introduction

Numerous studies have raised concerns about the quality of end-of-life (EOL) care provided to older adults in the US, with little change across 30 years of research.^1,2,3,4,5,6,7^ Many patients receive invasive treatments and services (eg, feeding tubes, dialysis) that are costly and do not significantly extend length of life or improve quality of life. Despite preferences for home, patients often die in hospitals and nursing homes, experiencing transitions from one health care setting of care to another late in life.^7,8,9,10,11,12,13,14,15,16^ Bereaved family members of Medicare beneficiaries highlight inadequacies of EOL care; 25% of decedents had inadequate pain management, 56% struggled with anxiety or depression, and more than 40% did not have their spiritual needs met.^5^ Medicare spending on patients in their last year of life accounts for 25% of program expenditures, with median spending in the last 6 months alone exceeding $25 000 in many parts of the country.^17,18^

Costly and aggressive EOL care has been linked to incentives embedded in traditional Medicare’s (TM) payment model. With half of Medicare beneficiaries now choosing Medicare Advantage (MA), the managed care alternative, we must understand how MA incentives may promote differences in EOL care utilization.^1,11^ MA plans receive fixed payments per enrollee that are independent of actual service use, and are protected from the costs of care associated with a hospice-eligible diagnosis once a beneficiary elects hospice. This combination of incentives may shift the location of care for beneficiaries with life-limiting illnesses (eg, moving from facility-based care through earlier transition to hospice or home health). On one hand, these incentives may induce MA plans to reduce expensive and potentially burdensome treatments, improving quality of care at the EOL.^19^ On the other hand, MA plans may limit access to expensive but beneficial services, creating challenges for patients and their families. If an MA enrollee elects hospice care, care for conditions other than the terminal, hospice-eligible diagnosis is covered through their MA plan, while hospice benefits shift to TM. This may be challenging for beneficiaries to navigate and lower enrollment in hospice.

We built on prior research that has compared MA and TM hospitalizations near the EOL by incorporating TM claims and MA encounter data to compare health care utilization across settings of care. Specifically, we compared potentially burdensome hospital treatments and hospitalizations, and postdischarge care for Medicare decedents in MA vs TM. We compared outcomes among all decedents as well as a subset who experienced one or more emergent hospitalizations in the last year of life with a life-limiting condition (cancer, dementia, or end-stage organ failure), a group with high care needs likely to benefit from care coordination, palliative care, or hospice referral.

## Methods

### Data and Study Sample

We used national Medicare claims data and newly available Medicare Advantage Encounter Data from 2016 to 2018 to gain a comprehensive picture of beneficiaries’ inpatient and postdischarge utilization near the EOL. Since 2008, MedPAR has included discharge abstracts for both MA and TM admissions for all Medicare-reimbursed hospitalizations. We supplemented these data with TM hospice files because hospice is a carve-out benefit for MA during the study period, and TM and MA home health files. This study of deceased individuals was deemed exempt from institutional review board review by the Colorado Multiple institutional review board.

The study sample comprised 20% of Medicare beneficiaries who are decedents 66 years and older with continuous enrollment in Parts A and B coverage in the last year of life who do not switch between TM or MA in the 6 months prior to death, with those who switch between the 2 followed in secondary analyses. We excluded patients residing in counties with less than 15% MA penetration because the experience of TM beneficiaries in markets that do not have a viable MA option may not generalize to those in markets with MA (eFigure 1 in Supplement 1 details our sample construction process).^20^

We constructed a subsample of decedents that experienced 1 or more emergency hospitalizations with a life-limiting condition present on the discharge abstract in the last year of life. Life-limiting conditions in our analysis included metastatic cancer, end-stage kidney disease, heart failure, liver failure, chronic obstructive pulmonary disease, and Alzheimer disease and related dementias (ADRD). Chronic conditions along with hospitalization via the emergency department are indicators of elevated mortality risk for older adults.^19,21^ Patients are less likely to experience long-term benefit from burdensome procedures or transfers and are well served by care coordination and palliative care following hospitalization. This subgroup would be more clinically homogenous than the full sample of decedents and could be eligible for referral to hospice care, which has the potential to be affected by different financial incentives between MA and TM plans.

### Study Outcomes

We first compared decedent-level measures of health care utilization in the last 6 months of life. We examined indicators of whether a patient experienced 1 or more potentially burdensome inpatient treatments, potentially burdensome hospitalizations, in-hospital death, and hospice care as part of their EOL utilization. Potentially burdensome treatments occur during hospitalization and included intubation/mechanical ventilation, tracheostomy, gastrostomy tube, enteral/parenteral nutrition, cardiopulmonary resuscitation, and hemodialysis.^10^ We examined 2 types of potentially burdensome hospitalizations that could be ascertained in MedPAR data: 3 or more hospitalizations in the last 90 days of life and 2 or more hospitalizations for pneumonia, urinary tract infection, dehydration, or sepsis in the last 120 days of life.^16^ We considered 3 measures of hospice use including late hospice transfers, occurring within the last 3 days of life, and 2 earlier transfers: transfers more than 3 days prior to death, and 30 or more days prior to death.

To understand mechanisms behind potential differences in the types of care used at the EOL in MA vs TM, we compared discharge destinations for each hospitalization discharge in the last 6 months of life. We first used MedPAR discharge destination codes to determine whether each hospitalization ended with in-hospital death and whether survivors were transferred to another hospital, received rehabilitative care in another facility, were discharged to facility-based hospice care, discharged to home hospice, discharged with home health care, or were sent home (including leaving against medical advice) without additional services. Finally, we used hospice and home health data to consider whether those discharged home receive timely home-based care, including home health and home hospice, within the first 3 days and first 7 days after hospitalization.^22^

### Analysis

We estimated linear probability regression models of patient- and hospitalization-level outcomes on an indicator of whether the decedent was enrolled in MA (vs TM) in the last 6 months of life, decedent demographic characteristics, Medicaid dual eligibility status, and indicators (fixed effects) for year of death and hospital referral region. Standard errors were clustered at the hospital referral region (HRR) level. We did not control for comorbid health conditions in the primary specifications because differences in coding intensity in MA encounter data vs TM claims raised concerns about whether this would introduce further bias.^23,24^ Regression coefficients represent the percentage point change in each outcome associated with being in MA relative to TM. Data were analyzed using Stata statistical software (version 18 MP; Stata Corp) with all analyses recreated between April 2024 and May 2024.

### Robustness Checks and Alternative Measures

While our primary models compared experiences of those who did not change coverage during the last 6 months of life, we were concerned that this could generate a misleading comparison if patients who were being denied care, for example, were moving between MA and TM near the EOL.^25,26^ Thus, we estimated intent-to-treat models, which assigned decedents to MA or TM based on the coverage they elected at the time 6 months prior to death—the beginning of the period frequently studied as the EOL. Despite recognized limitations about the quality of comorbidity data, we also examined models that included Elixhauser comorbidities from hospitalizations in the last year of life. Given concerns about nonuniform data quality reported by MA plans, we estimated models where the MA sample was limited to those enrolled in MA plans that have been shown to have high levels of reporting completeness by other researchers.^27^ Incomplete reporting could lead us to undercount home health visits among those discharged home.

We verified that our results were robust to using county rather than HRR fixed effects, and including hospital fixed effects (using the first hospital entered in the last 6 months of life for the decedent-level models). These within-hospital models allowed us to test whether use of different hospitals accounted for differences in postdischarge care. To compare patients treated in the same hospitals, we required hospitals to have at least 15% of their Medicare volume from MA and at least 15% from TM. All differences were considered statistically significant at a 2-sided *P* < .05 threshold, reflecting our large sample size.

## Results

Our sample included 659 135 TM decedents and 360 430 MA decedents (Table 1). Of those, 365 165 (55%) TM and 177 647 (49%) MA decedents experienced 1 or more emergency hospitalizations with a life-limiting condition in the last year of life. MA decedents were slightly younger than those with TM (82.5 years vs 83.3 years), on average, and less likely to be White (83% vs 86%). Patients with TM experienced more hospitalizations in the last 90 days of life (1.54 vs 1.35 in the life-limiting conditions group), and when hospitalized were more likely to be discharged to another acute or long-term hospital or skilled nursing facility (35% vs 30%), which could reflect differences in underlying health needs or preferences along with different practice styles and referral rules in MA (Table 2).

**Table 1.** Characteristics of Medicare Decedents aged 66 Years or Older, 2016 to 2018^a^

Characteristic	No. (%)			
	MA decedents (n = 360 430)	TM decedents (n = 659 135)	MA decedents with life-limiting illness (n = 177 647)	TM decedents with life-limiting illness (n = 365 165)
Decedent characteristics				
Age, mean (SD), y	82.5 (8.7)	83.3 (9.0)	81.8 (8.4)	82.9 (8.7)
Sex				
Female	190 199 (53)	357 581 (54)	91 453 (51)	195 838 (54)
Male	170 431 (47)	401 554 (46)	86 194 (49)	169 327 (46)
Race and ethnicity				
Black	39 359 (11)	56 554 (9)	22 988 (13)	36 590 (10)
Hispanic	9047(3)	10 480 (2)	4601 (3)	6171 (2)
White	299 157 (83)	569 031 (86)	143 894 (81)	309 916 (85)
Other^b^	14 561 (4)	23 070 (4)	6715 (4)	12 489 (3)
Dual-eligible	96 019 (27)	181 526 (28)	48 835 (27)	104 072 (29)
Hospitalization outcomes				
Inpatient death	70 176 (19)	146 592 (22)	49 919 (28)	109 002 (30)
Burdensome treatment	58 714 (16)	112 185 (17)	42 244 (24)	84 463 (23)
≥2 Infection hospitalizations	144 605 (40)	240 255 (36)	22 401 (13)	50 539 (14)
≥3 Hospitalizations, last 90 d	25 915 (7)	76 591 (12)	22 206 (13)	66 350 (18)
Hospitalized in last 6 mo of life	225 197 (62)	444 982 (68)	163 187 (92)	337 303 (92)
No. hospitalizations last 6 mo	1.2 (1.4)	1.7 (1.9)	1.9 (1.5)	2.5 (2.0)
Hospice outcomes				
Late hospice (≤3 d)	44 189 (12)	83 051 (13)	27 660 (16)	56 783 (16)
Hospice >3 d before death	154 300 (43)	273 343 (41)	72 853 (41)	144 569 (40)
Hospice ≥30 d before death	72 519 20)	127 213 (19)	28 477 (16)	55 724 (15)

Abbreviations: MA, Medicare Advantage; TM, traditional Medicare.

^a^ Decedents had A and B coverage for the last 12 months of life and lived in counties with at least 15% MA enrollment. Life-limiting sample includes those decedents with emergent, acute hospitalization with 1 or more life-limiting conditions (Alzheimer disease and related dementias, end-stage organ failure or cancer) present in the last 12 months of life.

^b^ Other includes Asian American, North American Native, missing, and unknown races and ethnicities.

**Table 2.** Characteristics of Posthospitalization Care, Discharges in the Last 6 Months of Life^a^

Characteristic	No (%)			
	MA decedents	TM decedents	MA decedents with life-limiting illness	TM decedents with life-limiting illness
Discharge outcome				
No. of discharges	398 669	834 301	312 840	682 372
In-hospital death	67 574 (17)	128 566 (15)	48 271 (15)	95 737 (14)
Hospital transfer	10 724 (3)	23 778 (3)	8040 (3)	18 629 (3)
Skilled nursing facility	114 817 (29)	291 755 (35)	92 882 (30)	246 950 (36)
Hospice facility	32 412 (8)	67 578 (8)	25 496 (8)	54 385 (8)
Home, no care	77 382 (19)	146 754 (18)	61 129 (20)	119 006 (17)
Home with home health	61 315 (15)	113 215 (14)	50 743 (16)	97 238 (14)
Home with home hospice	32 412 (8)	57 483 (7)	24 683 (8)	46 128 (7)
Follow-up if discharged home				
No. of discharges	171 104	317 451	136 556	262 370
Home hospice ≤3 d from discharge	32 630 (19)	57 300 (18)	24 594 (18)	45 547 (17)
Home hospice ≤7 d from discharge	36 976 (22)	64 887 (20)	27 994 (21)	51 687 (20)
Home health ≤3 d from discharge	34 597 (20)	52 538 (17)	26 574 (19)	41 979 (16)
Home health ≤7 d from discharge	41 407 (24)	60 951 (19)	32 036 (23)	48 932 (19)

Abbreviations: MA, Medicare Advantage; TM, traditional Medicare.

^a^ Decedents had A and B coverage for the last 12 months of life and lived in counties with at least 15% MA enrollment. Life-limiting sample includes those decedents with emergent, acute hospitalization with one or more life-limiting conditions (Alzheimer disease and related dementias, end-stage organ failure or cancer) present in the last 12 months of life. Discharges home include leaving against medical advice and all other discharges that did not include referrals to home health or facility-based care. Discharge destinations from MedPAR, hospice, and home health care use from Medicare claims and MA encounter data.

### Utilization in the Last 6 Months of Life

**Figure 1.**
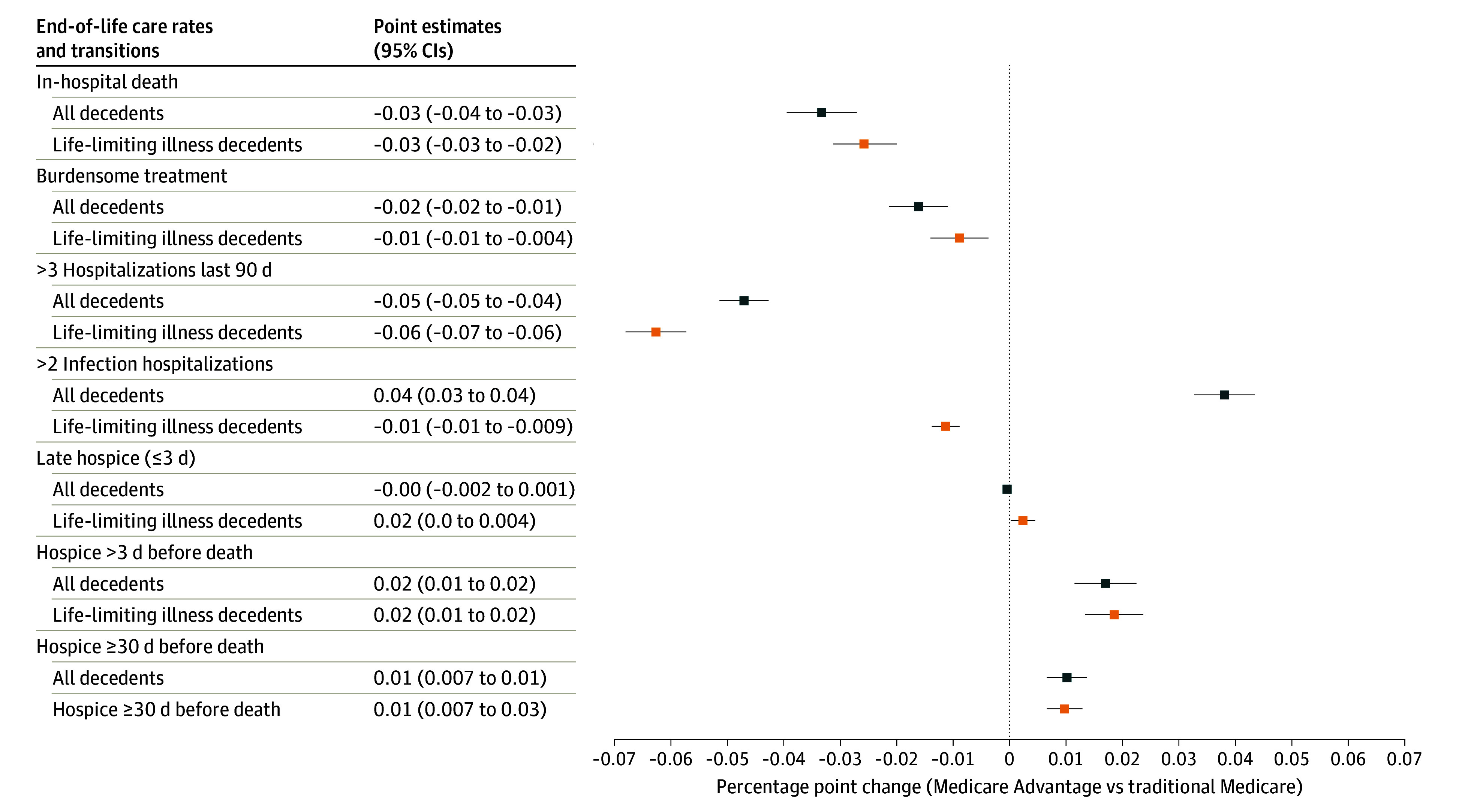
Regression-Adjusted Differences in Rates of Potentially Burdensome Treatments and Transitions Near the End of Life Among Medicare Advantage Enrollees vs Traditional Medicare, 2016 to 2018 Circles and squares represent the percentage point change associated with Medicare Advantage enrollment compared with traditional Medicare. Coefficients from regression models are adjusted for decedent demographics, year of death, and hospital referral region fixed effects. The 95% error bars are based on robust standard errors clustered at the HRR level.

After regression adjustment, we found that decedents in MA plans generally received less potentially burdensome EOL care (Figure 1; eTables A1-A2 in Supplement 1). MA enrollees were less likely to receive potentially burdensome treatments (adjusted difference −1.6 percentage points [pp], 95% CI, −2.1 to −1.1]), and less likely to have 3 or more hospitalizations in last 90 days of life (−4.7 pp; 95% CI, −5.1 to −4.3], but more likely to have 2 or more hospitalizations for infections in the last 120 days (3.8 pp; 95% CI, 3.3-4.4). Overall, MA decedents were less likely to die in the hospital (−3.3 pp; 95% CI, − 4.0 to −2.7). There was no difference in rates of late hospice transfer (0.04 pp; 95% CI, −0.2 to 0.1), though MA decedents were somewhat more likely to be moved to hospice for more than 3 days (1.7 pp; 95% CI, 1.1-2.0)] and at least 30 days (1.0 pp; 95% CI, 0.7-1.4) prior to death. Patterns were generally similar in the life-limiting conditions sample, though the difference in in-hospital death was smaller (−2.6 pp; 95% CI, −3.1 to −2.0) and hospitalizations for infections in the last 120 days were less common for MA decedents (−1.1 pp; 95% CI, −1.4 to −0.89).

### Discharge Destinations

**Figure 2.**
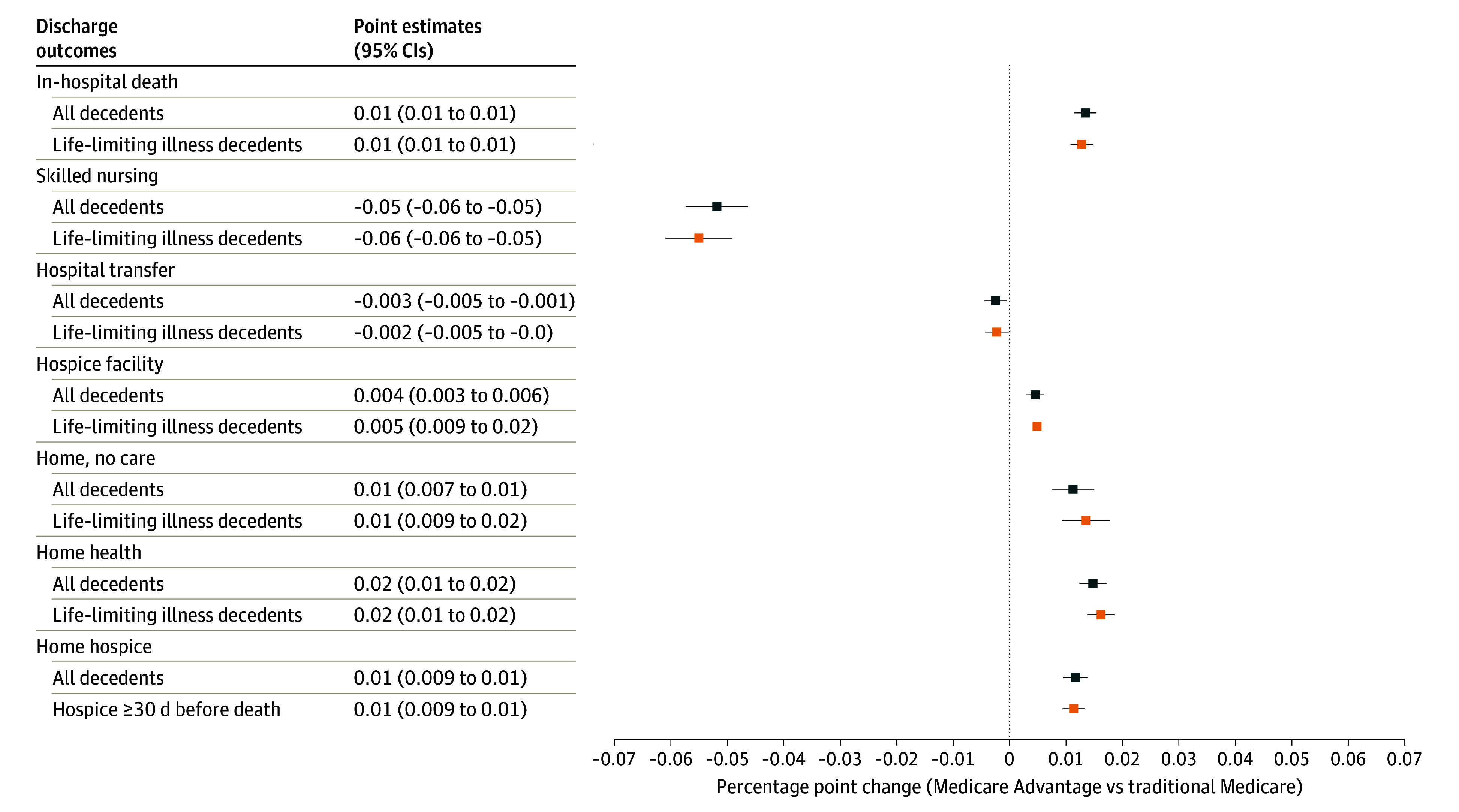
Regression-Adjusted Differences in Discharge Outcomes Among Medicare Advantage Enrollees vs Traditional Medicare Enrollees Hospitalized in the Last 6 Months of Life, 2016 to 2018 Circles and squares represent the percentage point change associated with Medicare Advantage enrollment compared with traditional Medicare. Coefficients from regression models are adjusted for decedent demographics, year of death, and hospital referral region fixed effects. the 95% error bars are based on robust standard errors clustered at the hospital referral region level. Mean values of each outcome are reported for all decedents/decedents with life-limiting illness.

654 007 Medicare beneficiaries experienced 1 232 970 hospitalizations in the last 6 months of life. Once hospitalized, MA decedents were more likely to die than those with TM (1.3 pp; 95% CI, 1.1-1.5; Figure 2). Among those discharged alive, MA enrollees were less likely to be transferred to a rehabilitative or skilled nursing care facility (adjusted difference, −5.2 pp; 95% CI, −5.7 to −46.0) and more likely to be sent home with no care (1.1 pp; 95% CI, 0.7-1.5), home health (1.5 pp; 95% CI, 1.2-1.7), or home hospice (1.2 pp; 95% CI, 0.9-1.4). Results were not significantly different when we restricted comparisons to patients with life-limiting illness (Figure 2; eTables A3-A4 in Supplement 1).

### Home Care

**Figure 3.**
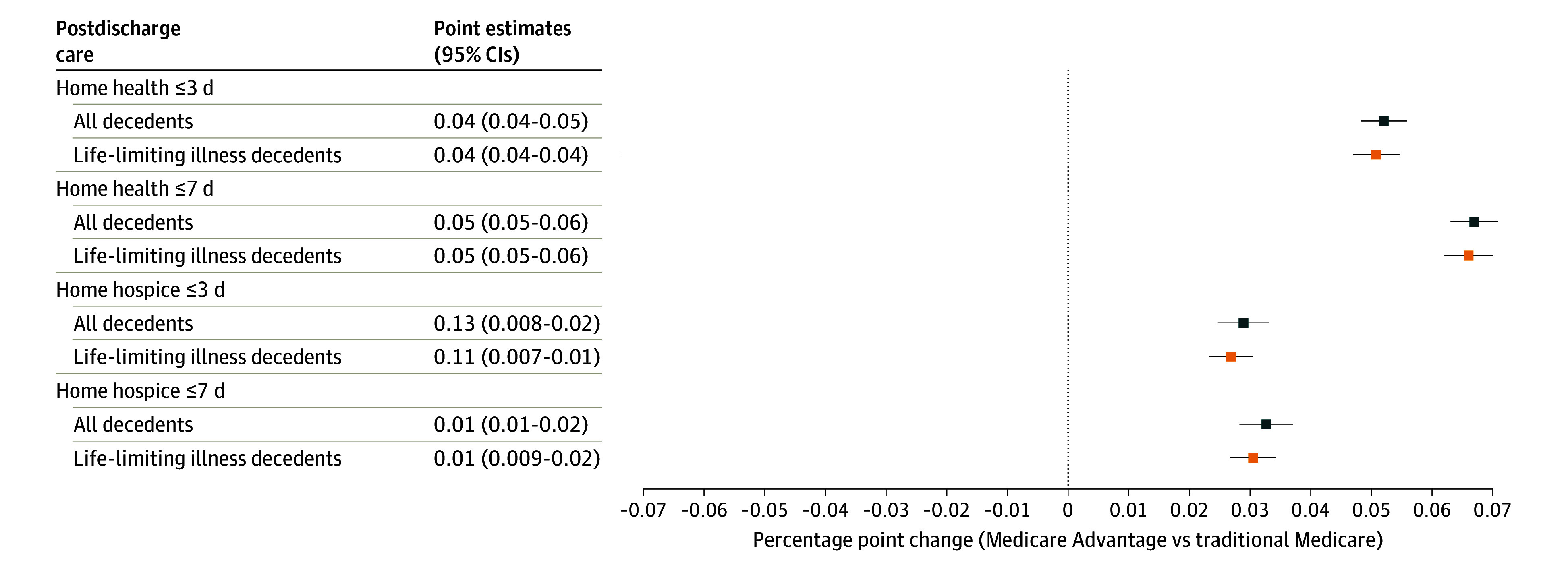
Regression-Adjusted Differences in Postdischarge Care Among Medicare Advantage vs traditional Medicare Hospitalized in the Last 6 Months of Life and Discharged Home, 2016 to 2018 Circles and squares represent the percentage point change associated with Medicare Advantage enrollment compared with traditional Medicare. Coefficients from regression models are adjusted for decedent demographics, year of death, and hospital referral region fixed effects. The 95% error bars are based on robust standard errors clustered at the hospital referral region level. Mean values of each outcome reported for all decedents/decedents with life-limiting illness.

When hospitalized patients were discharged home (488 555 discharges) in the last 6 months of life, MA decedents were more likely to receive home care in the first 3 days after discharge (home health: 4.2 pp; 95% CI, 0.04-0.05; home hospice: 1.3 pp 95% CI, 2.5-3.3), with similar results at 7 days (Figure 3). Findings were essentially unchanged for the life-limiting conditions sample and slightly larger in magnitude when we excluded MA plans with lower claim completeness (eTables A5-A6 in Supplement 1). The higher use of home care associated with MA among those discharged home implies that approximately half of the lower rate of discharge to skilled nursing in the MA group (Figure 2) is offset by higher rates of home health or home hospice.

### Robustness

We found similar results in our intent-to-treat models, which included those who switched between MA and TM in the last 6 months of life (eTables A7-A12 in Supplement 1). Differences between MA and TM were generally attenuated in these models, consistent with beneficiaries switching coverage type to access additional care. Results were also similar when we controlled for comorbid conditions, focused on MA plans with high completeness of encounter claims, and compared MA and TM decedents treated in the same hospitals.

## Discussion

In this national comparison of EOL health care utilization in MA vs TM, we found that MA enrollment was associated with lower rates of potentially burdensome hospitalizations and treatments, less skilled nursing facility care, and more home care after discharge. Financial incentives to reduce costs in MA appear to promote less use of potentially burdensome treatments but also leave some patients without home-based or facility care after hospitalization—higher rates of home care postdischarge offset roughly half of the difference in discharges to skilled nursing facilities. This potentially places a large burden on family caregivers or leaves patients in need of additional services. Although MA decedents experienced lower rates of dying in the hospital, the probability that an admission ended in death was higher for MA beneficiaries, which may reflect patients experiencing critical delays accessing acute hospital services, or MA plans doing a better job of keeping less sick patients out of the hospital. Although enrollment in MA has skyrocketed in recent years and the difference in health status between MA and TM beneficiaries is believed to have declined, we observed the same patterns of lower EOL health care utilization in MA that characterized previous decades.^7,28^

These results echo concerns raised by the Office of the Inspector General about coverage denials for skilled nursing and other important services in MA.^29^ We studied the experiences of a cohort of high-need patients who had 1 or more life-limiting medical conditions and required hospitalization through the ER at least once in the last year of life. These patients might especially benefit from the care coordination and home-based services that MA plans have the flexibility to offer, yet outcomes did not meaningfully differ relative to all decedents. Given these patterns, it is possible that family caregiving or out-of-pocket spending may have been required to fill this gap. While families may prefer to care for loved ones at home, this represents a valuable transfer from families to MA plans—median direct and indirect costs of ADRD caregiving, for example, are estimated at $150 000 over a 2-year period.^30^ This may represent an underappreciated area where payments to MA plans exceed the costs of care delivered and is concerning in light of research reporting lower satisfaction about the quality of EOL care among families of decedents with MA vs TM decedents, along with the broader debate around payments to MA plans.^31,32,33,34^

We found relatively low rates of hospice use in the life-limiting conditions sample; only 16% of MA and 15% of TM decedents received hospice care for 30 or more days prior to death, rates that were lower than use among all decedents. Overall, 16% of both groups experienced late hospice transitions 3 or fewer days prior to death. Similar proportions of decedents received any hospice care in the MA (43%) and TM (41%) groups, a figure that was not substantively different when we restricted to the life-limiting conditions sample. It is not possible to ascertain the impact of patient preferences, but the low utilization rate of hospice care suggests opportunities to improve EOL care, such as offering palliative care outside of hospice care.

### Limitations

Our results should be interpreted in light of several limitations. Beneficiaries’ choices of MA vs TM are nonrandom, and important determinants of the intensity of EOL care, including patient and family preferences and complete medical histories cannot be observed in claims data, could be associated with preferences over health plans. Our results should be interpreted as descriptions of differences in the care of MA and TM beneficiaries. However, we purposedly selected a group of patients that was more homogenous in their medical needs toward EOL to minimize the confounding effect of favorable selection into MA plans.^34^ Although we cannot test all possible sources of differences between TM and MA beneficiaries, we observed similar patterns when we compared increasingly clinically homogenous cohorts (ie, decedents with emergency hospitalization), when we accounted for hospital-assessed comorbid conditions, and when we compared patients treated at the same hospitals (ie, hospital fixed-effects models). We used the look-back method to identify decedents, which is subject to the criticism that it is much easier to identify decedents after the fact than predict death a priori and life-sustaining treatments would be appropriate for patients who did not appear close to the EOL when hospitalized.^35^ However, we partially address this problem by studying a sample with life-limiting conditions, which is determined based on an event that could shape future care in addition to death after this admission.

MA plans can choose which hospitals to contract with and potentially drive their enrollees to hospitals that are better at coordinating care, referring to hospice when appropriate or have a practice culture that aligns with the financial incentives of MA. However, MA vs TM differences persisted when we compared decedents using the same hospitals. It is possible that the difference in discharges to skilled nursing facilities resulted from MA plans providing additional supplemental benefits that enable beneficiaries to remain at home, which do not get recorded in the encounter data. Although MA plans are increasingly gaining approval to offer services such as palliative care and social home care benefits, these were not generally recognized as MA benefits during our study period. Whether the lower rates of posthospitalization care in MA reflect patient preferences or difficulties accessing care, it will be important to determine whether MA enrollees disproportionately rely on informal caregivers.

## Conclusions

This cross-sectional analysis of health care utilization in the last 6 months of life found that MA enrollment was associated with lower levels of facility-based care, including potentially burdensome treatments and admissions, and slightly higher rates of hospice use. Additional home care partially offset lower rates of skilled nursing for those with hospitalizations in the last year of life. Thus, our findings suggest that MA plans have the potential to reduce burdensome treatments for patients approching the EOL, although it is not possible to establish if these findings align with patients’ preferences or provide appropriate care. In addition, the higher rates of discharge home after hospitalization may shift care burdens to informal caregivers if Medicare beneficiaries enrolled in MA plans lack adequate formal support services after a hospitalization.

## Notes

Supplement 1. eFigure. Sample Construction eTable 1. Regression-adjusted Differences in Rates of Potentially Burdensome Treatments and Transitions Near the End-of-Life Among Medicare Advantage Enrollees vs. Traditional Medicare, 2016 – 2018 (Figure 1) eTable 2. Regression-adjusted Differences in Rates of Potentially Burdensome Treatments and Transitions Near the End-of-Life Among Medicare Advantage Enrollees vs. Traditional Medicare, 2016 – 2018: Life-limiting conditions sample (Figure 1) eTable 3. Regression-adjusted Differences in Discharge Outcomes Among Medicare Advantage Enrollees vs. Traditional Medicare Hospitalized in the 6 Months of Life, 2016 – 2018 (Figure 2) eTable 4. Regression-adjusted Differences in Discharge Outcomes Among Medicare Advantage Enrollees vs. Traditional Medicare Hospitalized in the 6 Months of Life, 2016 – 2018 Life-Limiting Conditions Sample (Figure 2) eTable 5. Regression-Adjusted Differences in Post-Discharge Care Among Medicare Advantage versus Traditional Medicare Enrollees Hospitalized in the Last 6 Months of Life and Discharged Home, 2016 - 2018 (Figure 3) eTable 6. Regression-Adjusted Differences in Post-Discharge Care Among Medicare Advantage versus Traditional Medicare Enrollees Hospitalized in the Last 6 Months of Life and Discharged Home, 2016 – 2018, Life-Limiting Conditions Sample (Figure 3) eTable 7. Regression-adjusted Differences in Rates of Potentially Burdensome Treatments and Transitions Near the End-of-Life Among Medicare Advantage Enrollees vs. Traditional Medicare, 2016 – 2018: Alternative Specifications and Samples eTable 8. Regression-adjusted Differences in Rates of Potentially Burdensome Treatments and Transitions Near the End-of-Life Among Medicare Advantage Enrollees vs. Traditional Medicare, 2016 – 2018: Alternative Specifications, Life-Limiting Conditions Sample eTable 9. Regression-adjusted Differences in Discharge Outcomes Among Medicare Advantage Enrollees vs. Traditional Medicare Hospitalized in the 6 Months of Life, 2016 – 2018, Alternative Samples and Specifications eTable 10. Regression-adjusted Differences in Discharge Outcomes Among Medicare Advantage Enrollees vs. Traditional Medicare Hospitalized in the 6 Months of Life, 2016 – 2018 Life-Limiting Conditions Sample Alternative Samples and Specifications eTable 11. Regression-Adjusted Differences in Post-Discharge Care Among Medicare Advantage versus Traditional Medicare Enrollees Hospitalized in the Last 6 Months of Life and Discharged Home, 2016 – 2018, Alternative Samples and Specifications eTable 12. Regression-Adjusted Differences in Post-Discharge Care Among Medicare Advantage versus Traditional Medicare Enrollees Hospitalized in the Last 6 Months of Life and Discharged Home, 2016 – 2018, Alternative Samples and Specifications, Life-Limiting Conditions Sample

Supplement 2. Data Sharing Statement
