# Supplementary material for: Medicare-Covered Services Near the End of Life in Medicare Advantage vs Traditional Medicare
Source: JAMA Health Forum. 2024 Jul 19;5(7):e241777. doi: 10.1001/jamahealthforum.2024.1777 (PMC11259900; doi:10.1001/jamahealthforum.2024.1777)
Supplement: Supplement 1. — eFigure. Sample Construction eTable 1. Regression-adjusted Differences in Rates of Potentially Burdensome Treatments and Transitions Near the End-of-Life Among Medicare Advantage Enrollees vs. Traditional Medicare, 2016 – 2018 (Figure 1) eTable 2. Regression-adjusted Differences in Rates of Potentially Burdensome Treatments and Transitions Near the End-of-Life Among Medicare Advantage Enrollees vs. Traditional Medicare, 2016 – 2018: Life-limiting conditions sample (Figure 1) eTable 3. Regression-adjusted Differences in Discharge Outcomes Among Medicare Advantage Enrollees vs. Traditional Medicare Hospitalized in the 6 Months of Life, 2016 – 2018 (Figure 2) eTable 4. Regression-adjusted Differences in Discharge Outcomes Among Medicare Advantage Enrollees vs. Traditional Medicare Hospitalized in the 6 Months of Life, 2016 – 2018 Life-Limiting Conditions Sample (Figure 2) eTable 5. Regression-Adjusted Differences in Post-Discharge Care Among Medicare Advantage versus Traditional Medicare Enrollees Hospitalized in the Last 6 Months of Life and Discharged Home, 2016 - 2018 (Figure 3) eTable 6. Regression-Adjusted Differences in Post-Discharge Care Among Medicare Advantage versus Traditional Medicare Enrollees Hospitalized in the Last 6 Months of Life and Discharged Home, 2016 – 2018, Life-Limiting Conditions Sample (Figure 3) eTable 7. Regression-adjusted Differences in Rates of Potentially Burdensome Treatments and Transitions Near the End-of-Life Among Medicare Advantage Enrollees vs. Traditional Medicare, 2016 – 2018: Alternative Specifications and Samples eTable 8. Regression-adjusted Differences in Rates of Potentially Burdensome Treatments and Transitions Near the End-of-Life Among Medicare Advantage Enrollees vs. Traditional Medicare, 2016 – 2018: Alternative Specifications, Life-Limiting Conditions Sample eTable 9. Regression-adjusted Differences in Discharge Outcomes Among Medicare Advantage Enrollees vs. Traditional Medicare Hospitalized in the 6 Months of Life [file jamahealthforum-e241777-s001.pdf]

Supplemental Online Content

Nicholas LH, Fischer SM, Arbaje AI, et al. Medicare-covered services near the end of life in Medicare Advantage vs traditional Medicare. *JAMA Health Forum*. 2024;5(7):e241777. doi:10.1001/jamahealthforum.2024.1777

eFigure. Sample Construction
eTable 1. Regression-adjusted Differences in Rates of Potentially Burdensome Treatments and Transitions Near the End-of-Life Among Medicare Advantage Enrollees vs. Traditional Medicare, 2016 – 2018 (Figure 1)
eTable 2. Regression-adjusted Differences in Rates of Potentially Burdensome Treatments and Transitions Near the End-of-Life Among Medicare Advantage Enrollees vs. Traditional Medicare, 2016 – 2018: Life-limiting conditions sample (Figure 1)
eTable 3. Regression-adjusted Differences in Discharge Outcomes Among Medicare Advantage Enrollees vs. Traditional Medicare Hospitalized in the 6 Months of Life, 2016 – 2018 (Figure 2)
eTable 4. Regression-adjusted Differences in Discharge Outcomes Among Medicare Advantage Enrollees vs. Traditional Medicare Hospitalized in the 6 Months of Life, 2016 – 2018 Life-Limiting Conditions Sample (Figure 2)
eTable 5. Regression-Adjusted Differences in Post-Discharge Care Among Medicare Advantage versus Traditional Medicare Enrollees Hospitalized in the Last 6 Months of Life and Discharged Home, 2016 - 2018 (Figure 3)
eTable 6. Regression-Adjusted Differences in Post-Discharge Care Among Medicare Advantage versus Traditional Medicare Enrollees Hospitalized in the Last 6 Months of Life and Discharged Home, 2016 – 2018, Life-Limiting Conditions Sample (Figure 3)
eTable 7. Regression-adjusted Differences in Rates of Potentially Burdensome Treatments and Transitions Near the End-of-Life Among Medicare Advantage Enrollees vs. Traditional Medicare, 2016 – 2018: Alternative Specifications and Samples
eTable 8. Regression-adjusted Differences in Rates of Potentially Burdensome Treatments and Transitions Near the End-of-Life Among Medicare Advantage Enrollees vs. Traditional Medicare, 2016 – 2018: Alternative Specifications, Life-Limiting Conditions Sample
eTable 9. Regression-adjusted Differences in Discharge Outcomes Among Medicare Advantage Enrollees vs. Traditional Medicare Hospitalized in the 6 Months of Life, 2016 – 2018, Alternative Samples and Specifications
eTable 10. Regression-adjusted Differences in Discharge Outcomes Among Medicare Advantage Enrollees vs. Traditional Medicare Hospitalized in the 6 Months of Life, 2016 – 2018 Life-Limiting Conditions Sample Alternative Samples and Specifications
eTable 11. Regression-Adjusted Differences in Post-Discharge Care Among Medicare Advantage versus Traditional Medicare Enrollees Hospitalized in the Last 6 Months of Life and Discharged Home, 2016 – 2018, Alternative Samples and Specifications
eTable 12. Regression-Adjusted Differences in Post-Discharge Care Among Medicare Advantage versus Traditional Medicare Enrollees Hospitalized in the Last 6 Months of Life and Discharged Home, 2016 – 2018, Alternative Samples and Specifications, Life-Limiting Conditions Sample

© 2024 Nicholas LH et al. *JAMA Health Forum.*

This supplemental material has been provided by the authors to give readers additional information about their work.

© 2024 Nicholas LH et al. *JAMA Health Forum.*

eFigure. Sample Construction

1,197,202 deaths among Medicare beneficiaries aged 66+ living in 50 states and DC (20% sample group)

Drop 44,681 with incomplete A+B coverage during the study period

Drop 629 with missing zip code or missing Hospital Referral Region

Drop 104,444 with county MA penetration < 15%:

Intent to treat sample: N = 1,044,448

Drop 24,883 who switch between MA and TM in last 6 months of life:

Main Sample: N = 1,019565

Main sample members have **N = 1,232970 hospitalizations** in the last 6 months of life including **N = 488,555 discharges home**

eTable 1: Regression-adjusted Differences in Rates of Potentially Burdensome Treatments and Transitions Near the End-of-Life Among Medicare Advantage Enrollees vs. Traditional Medicare, 2016 – 2018 (Figure 1)

	(1) Died In Hospital	(2) Burdensome Treatment	(3) 2+ hosp. with pneumonia, sepsis, UTI or dehydration in last 120 days	(4) 3+ hospitalizations in the last 90 days of life	(5) Hospice Admission <=3 Days Before Death	(6) Hospice Admission > 3 days before death	(7) Hospice Admission > 30 days before death
MA	-0.033**	-0.016**	0.038**	-0.047**	-0.000	0.017**	0.010**
	[-0.040,-0.027]	[-0.021,-0.011]	[0.033,0.044]	[-0.051,-0.043]	[-0.002,0.001]	[0.011,0.023]	[0.007,0.014]
Age 75-85	-0.030**	-0.055**	-0.005**	-0.015**	0.016**	0.071**	0.042**
	[-0.033,-0.028]	[-0.058,-0.052]	[-0.008,-0.002]	[-0.017,-0.013]	[0.014,0.017]	[0.067,0.074]	[0.039,0.044]
Age 85+	-0.106**	-0.166**	0.071**	-0.062**	0.022**	0.154**	0.100**
	[-0.108,-0.104]	[-0.169,-0.163]	[0.066,0.075]	[-0.065,-0.060]	[0.019,0.024]	[0.146,0.161]	[0.095,0.105]
Female	-0.009**	-0.021**	0.013**	-0.007**	-0.005**	0.064**	0.044**
	[-0.011,-0.007]	[-0.023,-0.018]	[0.010,0.016]	[-0.009,-0.005]	[-0.007,-0.003]	[0.062,0.067]	[0.042,0.046]
Black	0.046**	0.092**	-0.050**	0.025**	-0.034**	-0.055**	-0.032**
	[0.042,0.051]	[0.086,0.097]	[-0.056,-0.044]	[0.022,0.028]	[-0.037,-0.031]	[-0.060,-0.050]	[-0.036,-0.028]
Hispanic	0.030**	0.049**	-0.032**	0.011**	-0.013**	-0.035**	-0.021**
	[0.023,0.038]	[0.041,0.057]	[-0.040,-0.023]	[0.007,0.016]	[-0.019,-0.008]	[-0.047,-0.022]	[-0.029,-0.014]
Other Race	0.059**	0.061**	-0.022**	0.008**	-0.016**	-0.069**	-0.042**
	[0.048,0.069]	[0.055,0.068]	[-0.030,-0.014]	[0.004,0.013]	[-0.020,-0.011]	[-0.083,-0.056]	[-0.049,-0.034]
Dual Eligible	-0.030**	-0.008*	0.066**	-0.011**	-0.013**	-0.002	0.020**
	[-0.038,-0.022]	[-0.015,-0.000]	[0.056,0.075]	[-0.016,-0.007]	[-0.015,-0.011]	[-0.010,0.006]	[0.015,0.025]
Observations	1019565	1019565	1019565	1019565	1019565	1019565	1019565

Models control for HRR and year fixed effects, standard errors clustered at the HRR level.

eTable 2: Regression-adjusted Differences in Rates of Potentially Burdensome Treatments and Transitions Near the End-of-Life Among Medicare Advantage Enrollees vs. Traditional Medicare, 2016 – 2018: Life-limiting conditions sample (Figure 1)

	(1)<br>Died In Hospital	(2)<br>Burdensome Treatment	(3)<br>2+ hosp. with pneumonia, sepsis, UTI or dehydration in last 120 days	(4)<br>3+ hospitalizations in the last 90 days of life	(5)<br>Hospice Admission <=3 Days Before Death	(6)<br>Hospice Admission > 3 days before death	(7)<br>Hospice Admission > 30 days before death
MA	-0.026**	-0.009**	-0.011**	-0.063**	0.002*	0.019**	0.010**
	[-0.031,-0.020]	[-0.014,-0.004]	[-0.014,-0.009]	[-0.068,-0.057]	[0.000,0.004]	[0.013,0.024]	[0.007,0.013]
Age 75-85	-0.047**	-0.081**	0.006**	-0.030**	0.012**	0.046**	0.026**
	[-0.050,-0.043]	[-0.086,-0.077]	[0.003,0.008]	[-0.033,-0.027]	[0.009,0.014]	[0.042,0.050]	[0.023,0.029]
Age 85+	-0.122**	-0.214**	0.019**	-0.092**	0.018**	0.110**	0.063**
	[-0.125,-0.118]	[-0.218,-0.210]	[0.016,0.022]	[-0.095,-0.089]	[0.015,0.021]	[0.104,0.117]	[0.059,0.067]
Female	-0.010**	-0.031**	-0.000	-0.011**	-0.009**	0.048**	0.029**
	[-0.013,-0.007]	[-0.034,-0.028]	[-0.002,0.002]	[-0.014,-0.009]	[-0.011,-0.006]	[0.045,0.051]	[0.027,0.031]
Black	0.046**	0.115**	-0.011**	0.026**	-0.044**	-0.034**	-0.011**
	[0.041,0.052]	[0.107,0.123]	[-0.015,-0.007]	[0.022,0.031]	[-0.048,-0.039]	[-0.039,-0.028]	[-0.015,-0.007]
Hispanic	0.028**	0.063**	-0.019**	0.014**	-0.008	-0.001	-0.003
	[0.016,0.039]	[0.050,0.077]	[-0.026,-0.012]	[0.007,0.022]	[-0.018,0.001]	[-0.012,0.009]	[-0.010,0.005]
Other Race	0.072**	0.084**	-0.011**	0.014**	-0.016**	-0.050**	-0.024**
	[0.058,0.085]	[0.076,0.093]	[-0.016,-0.005]	[0.008,0.021]	[-0.022,-0.009]	[-0.064,-0.035]	[-0.030,-0.017]
Dual Eligible	-0.027**	-0.001	0.046**	-0.021**	-0.021**	0.001	0.019**
	[-0.034,-0.019]	[-0.008,0.006]	[0.043,0.049]	[-0.026,-0.015]	[-0.024,-0.018]	[-0.007,0.008]	[0.015,0.023]
Observations	542812	542812	542812	542812	542812	542812	542812

Models control for HRR and year fixed effects, standard errors clustered at the HRR level. Life-limiting conditions include ADRD, end-stage organ failure, cancer.

eTable 3: Regression-adjusted Differences in Discharge Outcomes Among Medicare Advantage Enrollees vs. Traditional Medicare Hospitalized in the 6 Months of Life, 2016 – 2018 (Figure 2)

	(1)<br>In-Hospital Death	(2)<br>Hospital Transfer	(3)<br>Skilled Nursing Facility	(4)<br>Hospice Facility	(5)<br>Home No Care	(6)<br>Home Health	(7)<br>Home Hospice
MA	0.013**	-0.003*	-0.052**	0.004**	0.011**	0.015**	0.012**
	[0.011,0.015]	[-0.005,-0.001]	[-0.057,-0.046]	[0.003,0.006]	[0.007,0.015]	[0.012,0.017]	[0.009,0.014]
Age 75-85	-0.010**	-0.007**	0.073**	0.012**	-0.067**	-0.007**	0.007**
	[-0.012,-0.009]	[-0.008,-0.006]	[0.070,0.076]	[0.010,0.013]	[-0.069,-0.064]	[-0.009,-0.005]	[0.006,0.008]
Age 85+	-0.029**	-0.022**	0.143**	0.031**	-0.123**	-0.024**	0.022**
	[-0.031,-0.026]	[-0.024,-0.021]	[0.138,0.149]	[0.029,0.034]	[-0.126,-0.119]	[-0.027,-0.020]	[0.020,0.025]
Female	-0.002*	-0.004**	0.009**	0.007**	-0.022**	0.005**	0.008**
	[-0.003,-0.000]	[-0.005,-0.003]	[0.007,0.011]	[0.006,0.009]	[-0.023,-0.020]	[0.004,0.007]	[0.006,0.009]
Black	0.006**	-0.003**	-0.015**	-0.017**	0.006**	0.022**	0.002
	[0.003,0.008]	[-0.005,-0.001]	[-0.021,-0.009]	[-0.020,-0.015]	[0.002,0.010]	[0.017,0.027]	[-0.000,0.004]
Hispanic	0.013**	0.001	-0.112**	-0.009**	0.056**	0.032**	0.021**
	[0.008,0.018]	[-0.001,0.003]	[-0.127,-0.097]	[-0.014,-0.004]	[0.049,0.064]	[0.020,0.044]	[0.015,0.026]
Other Race	0.038**	0.002	-0.066**	-0.008**	0.021**	0.013**	0.002
	[0.033,0.043]	[-0.000,0.003]	[-0.079,-0.052]	[-0.012,-0.005]	[0.014,0.029]	[0.007,0.019]	[-0.005,0.008]
Dual Eligible	-0.020**	-0.006**	0.140**	0.002	-0.056**	-0.036**	-0.024**
	[-0.023,-0.018]	[-0.007,-0.005]	[0.134,0.146]	[-0.000,0.004]	[-0.060,-0.052]	[-0.040,-0.032]	[-0.026,-0.021]
Observations	1232970	1232970	1232970	1232970	1232970	1232970	1232970

Models control for HRR and year fixed effects, standard errors clustered at the HRR level.

eTable 4: Regression-adjusted Differences in Discharge Outcomes Among Medicare Advantage Enrollees vs. Traditional Medicare Hospitalized in the 6 Months of Life, 2016 – 2018 Life-Limiting Conditions Sample (Figure 2)

	(1) In-Hospital Death	(2) Hospital Transfer	(3) Skilled Nursing Facility	(4) Hospice Facility	(5) Home No Care	(6) Home Health	(7) Home Hospice
MA	0.013**	-0.002*	-0.055**	0.005**	0.013**	0.016**	0.011**
	[0.011,0.015]	[-0.005,-0.000]	[-0.061,-0.049]	[0.003,0.006]	[0.009,0.018]	[0.014,0.019]	[0.009,0.013]
Age 75-85	-0.008**	-0.007**	0.075**	0.011**	-0.068**	-0.008**	0.006**
	[-0.010,-0.007]	[-0.008,-0.006]	[0.071,0.078]	[0.010,0.013]	[-0.071,-0.065]	[-0.011,-0.006]	[0.005,0.007]
Age 85+	-0.022**	-0.021**	0.142**	0.030**	-0.123**	-0.027**	0.021**
	[-0.024,-0.020]	[-0.023,-0.020]	[0.136,0.147]	[0.028,0.033]	[-0.127,-0.119]	[-0.031,-0.023]	[0.019,0.023]
Female	-0.001	-0.004**	0.009**	0.006**	-0.022**	0.007**	0.007**
	[-0.003,0.000]	[-0.005,-0.003]	[0.006,0.011]	[0.005,0.007]	[-0.024,-0.020]	[0.005,0.009]	[0.006,0.009]
Black	0.007**	-0.003**	-0.017**	-0.017**	0.008**	0.021**	0.002
	[0.004,0.010]	[-0.005,-0.001]	[-0.023,-0.010]	[-0.020,-0.015]	[0.003,0.012]	[0.016,0.026]	[-0.000,0.005]
Hispanic	0.007*	0.000	-0.111**	-0.008**	0.058**	0.035**	0.020**
	[0.002,0.012]	[-0.002,0.002]	[-0.128,-0.094]	[-0.014,-0.003]	[0.050,0.066]	[0.022,0.047]	[0.014,0.027]
Other Race	0.031**	0.001	-0.063**	-0.009**	0.023**	0.017**	0.002
	[0.026,0.035]	[-0.001,0.003]	[-0.079,-0.048]	[-0.013,-0.005]	[0.015,0.031]	[0.010,0.024]	[-0.004,0.009]
Dual Eligible	-0.013**	-0.006**	0.137**	0.002	-0.056**	-0.041**	-0.024**
	[-0.015,-0.011]	[-0.007,-0.005]	[0.131,0.143]	[-0.000,0.004]	[-0.060,-0.053]	[-0.045,-0.037]	[-0.026,-0.021]
Observations	995212	995212	995212	995212	995212	995212	995212

Models control for HRR and year fixed effects, standard errors clustered at the HRR level. Life-limiting conditions include ADRD, end-stage organ failure, cancer.

eTable 5: Regression-Adjusted Differences in Post-Discharge Care Among Medicare Advantage versus Traditional Medicare Enrollees Hospitalized in the Last 6 Months of Life and Discharged Home, 2016 - 2018 (Figure 3)

	(1)<br>Home Health <=<br>3 Days Post-<br>Discharge	(2)<br>Home Health <=<br>7 Days Post-<br>Discharge	(3)<br>Home Hospice<br><= 3 Days Post-<br>Discharge	(4)<br>Home Hospice<br><= 7 Days Post-<br>Discharge		
MA	0.042**	0.055**	0.013**	0.015**		
	[0.038,0.046]	[0.051,0.059]	[0.008,0.017]	[0.010,0.020]		
Age 75-85	0.037**	0.041**	0.045**	0.047**		
	[0.035,0.040]	[0.038,0.044]	[0.042,0.047]	[0.044,0.050]		
Age 85+	0.095**	0.104**	0.125**	0.134**		
	[0.091,0.099]	[0.100,0.108]	[0.119,0.131]	[0.128,0.140]		
Female	0.020**	0.023**	0.025**	0.028**		
	[0.018,0.022]	[0.020,0.025]	[0.023,0.028]	[0.026,0.031]		
Black	-0.015**	-0.017**	-0.021**	-0.026**		
	[-0.019,-0.012]	[-0.021,-0.013]	[-0.025,-0.016]	[-0.031,-0.021]		
Hispanic	-0.009*	-0.009*	-0.003	-0.004		
	[-0.017,-0.001]	[-0.018,-0.001]	[-0.010,0.005]	[-0.012,0.004]		
Other Race	-0.009**	-0.010**	-0.016**	-0.018**		
	[-0.015,-0.002]	[-0.018,-0.003]	[-0.025,-0.006]	[-0.028,-0.008]		
Dual Eligible	-0.014**	-0.016**	-0.010**	-0.012**		
	[-0.019,-0.010]	[-0.021,-0.012]	[-0.017,-0.004]	[-0.019,-0.006]		
Observations	488555	488555	488555	488555	488555	488555

Models control for HRR and year fixed effects, standard errors clustered at the HRR level. Timing of visits identified using revenue center codes.

eTable 6: Regression-Adjusted Differences in Post-Discharge Care Among Medicare Advantage versus Traditional Medicare Enrollees Hospitalized in the Last 6 Months of Life and Discharged Home, 2016 – 2018, Life-Limiting Conditions Sample (Figure 3)

	(1)<br>Home Health <= 3 Days Post-Discharge	(2)<br>Home Health <= 7 Days Post-Discharge	(3)<br>Home Hospice <= 3 Days Post-Discharge	(4)<br>Home Hospice <= 7 Days Post-Discharge
MA	0.041**	0.055**	0.011**	0.013**
	[0.037,0.045]	[0.050,0.059]	[0.007,0.015]	[0.009,0.017]
Age 75-85	0.037**	0.041**	0.042**	0.044**
	[0.034,0.039]	[0.038,0.044]	[0.039,0.045]	[0.041,0.047]
Age 85+	0.092**	0.101**	0.119**	0.128**
	[0.088,0.096]	[0.097,0.105]	[0.114,0.125]	[0.122,0.134]
Female	0.019**	0.022**	0.024**	0.026**
	[0.017,0.021]	[0.019,0.024]	[0.021,0.026]	[0.024,0.029]
Black	-0.015**	-0.016**	-0.020**	-0.025**
	[-0.018,-0.011]	[-0.020,-0.012]	[-0.025,-0.015]	[-0.030,-0.020]
Hispanic	-0.010*	-0.010	-0.003	-0.003
	[-0.019,-0.001]	[-0.020,0.001]	[-0.012,0.007]	[-0.013,0.006]
Other Race	-0.008*	-0.010**	-0.014**	-0.017**
	[-0.014,-0.002]	[-0.017,-0.003]	[-0.023,-0.006]	[-0.026,-0.008]
Dual Eligible	-0.015**	-0.017**	-0.011**	-0.012**
	[-0.019,-0.011]	[-0.022,-0.013]	[-0.016,-0.005]	[-0.018,-0.006]
Observations	398926	398926	398926	398926

Models control for HRR and year fixed effects, standard errors clustered at the HRR level. Life-limiting conditions include ADRD, end-stage organ failure, and cancer. Timing of visits identified using revenue center codes.

eTable 7: Regression-adjusted Differences in Rates of Potentially Burdensome Treatments and Transitions Near the End-of-Life Among Medicare Advantage Enrollees vs. Traditional Medicare, 2016 – 2018: Alternative Specifications and Samples

	(1) Died In Hospital	(2) Burdensome Treatment	(3) 2+ hosp. with pneumonia, sepsis, UTI or dehydration in last 120 days	(4) 3+ hospitalizations in the last 90 days of life	(5) Hospice Admission <=3 Days Before Death	(6) Hospice Admission > 3 days before death	(7) Hospice Admission > 30 days before death
MA: Main Sample	-0.033**	-0.016**	0.038**	-0.047**	-0.000	0.017**	0.010**
	[-0.040,-0.027]	[-0.021,-0.011]	[0.033,0.044]	[-0.051,-0.043]	[-0.002,0.001]	[0.011,0.023]	[0.007,0.014]
MA: Intent to Treat	-0.032**	-0.015**	0.034**	-0.044**	-0.000	0.018**	0.010**
Sample	[-0.038,-0.026]	[-0.020,-0.010]	[-0.020,-0.014]	[-0.048,-0.040]	[-0.002,0.001]	[0.012,0.023]	[0.007,0.014]
MA: Main with	-0.016**	0.001	-0.017**	-0.050**	0.005**	0.012**	0.004*
Comorbidities	[-0.021,-0.012]	[-0.003,0.005]	[0.033,0.044]	[-0.054,-0.045]	[0.003,0.008]	[0.008,0.016]	[0.001,0.006]
MA: Complete	-0.027**	-0.007**	0.019**	-0.044**	0.005**	0.031**	0.018**
Contracts Sample	[-0.031,-0.023]	[-0.010,-0.004]	[0.013,0.024]	[-0.047,-0.041]	[0.003,0.007]	[0.025,0.037]	[0.014,0.022]

Sample sizes for alternative comparison groups: main sample: 1019565, intent to treat (includes switchers, MA status assigned 6 months prior to death): 1044448, complete contracts: 1016005. Elixhauser comorbidities are ascertained using hospitalizations in the last year of life. 35% of the sample does not have a hospitalization during this period. Since their comorbidities cannot be accurately assigned, we do not recommend interpreting the comorbidity models in the main sample, though we report it for completeness.

eTable 8: Regression-adjusted Differences in Rates of Potentially Burdensome Treatments and Transitions Near the End-of-Life Among Medicare Advantage Enrollees vs. Traditional Medicare, 2016 – 2018: Alternative Specifications, Life-Limiting Conditions Sample

	(1) Died In Hospital	(2) Burdensome Treatment	(3) 2+ hosp. with pneumonia, sepsis, UTI or dehydration in last 120 days	(4) 3+ hospitalizations in the last 90 days of life	(5) Hospice Admission <=3 Days Before Death	(6) Hospice Admission > 3 days before death	(7) Hospice Admission > 30 days before death
MA: Main Sample	-0.026**	-0.009**	-0.011**	-0.063**	0.002*	0.019**	0.010**
	[-0.031,-0.020]	[-0.014,-0.004]	[-0.014,-0.009]	[-0.068,-0.057]	[0.000,0.004]	[0.013,0.024]	[0.007,0.013]
MA: Intent to Treat	-0.025**	-0.008**	-0.011**	-0.058**	0.002*	0.019**	0.009**
Sample	[-0.030,-0.020]	[-0.013,-0.003]	[-0.014,-0.009]	[-0.063,-0.053]	[0.000,0.004]	[0.014,0.024]	[0.006,0.013]
MA: Add	-0.016**	0.001	-0.017**	-0.050**	0.005**	0.012**	0.004*
Comorbidities	[-0.021,-0.012]	[-0.003,0.005]	[0.033,0.044]	[-0.054,-0.045]	[0.003,0.008]	[0.008,0.016]	[0.001,0.006]
MA: Complete	-0.027**	-0.008**	-0.012**	-0.064**	0.005**	0.027**	0.013**
Contracts Sample	[-0.032,-0.023]	[-0.011,-0.004]	[-0.015,-0.009]	[-0.068,-0.059]	[0.003,0.007]	[0.021,0.032]	[0.010,0.017]

Sample sizes for alternative comparison groups: main sample: 542812, intent to treat (includes switchers, MA status assigned 6 months prior to death): 557672, complete contracts: 534252.

eTable 9: Regression-adjusted Differences in Discharge Outcomes Among Medicare Advantage Enrollees vs. Traditional Medicare Hospitalized in the 6 Months of Life, 2016 – 2018, Alternative Samples and Specifications

	(1) In-Hospital Death	(2) Hospital Transfer	(3) Skilled Nursing Facility	(4) Hospice Facility	(5) Home No Care	(6) Home Health	(7) Home Hospice
MA: Main Sample	0.013**	-0.003*	-0.052**	0.004**	0.011**	0.015**	0.012**
	[0.011,0.015]	[-0.005,-0.001]	[-0.057,-0.046]	[0.003,0.006]	[0.007,0.015]	[0.012,0.017]	[0.009,0.014]
MA: Intent to	0.011**	-0.002*	-0.044**	0.004**	0.009**	0.012**	0.011**
Treat	[0.009,0.013]	[-0.004,-0.0004]	[-0.049,-0.039]	[0.002,0.006]	[0.006,0.013]	[0.010,0.015]	[0.008,0.013]
MA: Add	0.004**	-0.004**	-0.037**	0.006**	0.008**	0.015**	0.009**
Comorbidities	[0.002,0.006]	[-0.005,-0.003]	[-0.042,-0.033]	[0.004,0.007]	[0.004,0.012]	[0.012,0.017]	[0.007,0.010]
MA: Complete	0.012**	-0.005**	-0.048**	0.006**	0.010**	0.014**	0.011**
Contracts Sample	[0.010,0.015]	[-0.006,-0.004]	[-0.053,-0.042]	[0.005,0.008]	[0.006,0.014]	[0.011,0.017]	[0.009,0.012]
Hospital Fixed	0.011**	-0.000	-0.048**	0.003**	0.014**	0.012**	0.010**
Effects	[0.009,0.013]	[-0.001,0.001]	[-0.051,-0.045]	[0.002,0.004]	[0.011,0.016]	[0.010,0.014]	[0.008,0.011]

Sample sizes for alternative comparison groups: main sample: 1232970, intent to treat (includes switchers, MA status assigned 6 months prior to death): 1267836, complete contracts: 1073453, hospital fixed effects: 1059971.

eTable 10: Regression-adjusted Differences in Discharge Outcomes Among Medicare Advantage Enrollees vs. Traditional Medicare Hospitalized in the 6 Months of Life, 2016 – 2018 Life-Limiting Conditions Sample Alternative Samples and Specifications

	(1)<br>In-Hospital Death	(2)<br>Hospital Transfer	(3)<br>Skilled Nursing Facility	(4)<br>Hospice Facility	(5)<br>Home No Care	(6)<br>Home Health	(7)<br>Home Hospice
MA	0.013**	-0.002*	-0.055**	0.005**	0.013**	0.016**	0.011**
	[0.011,0.015]	[-0.005,-0.000]	[-0.061,-0.049]	[0.003,0.006]	[0.009,0.018]	[0.014,0.019]	[0.009,0.013]
MA: Intent to	0.010**	-0.002*	-0.046**	0.004**	0.011**	0.013**	0.010**
Treat	[0.009,0.012]	[-0.004,-0.002]	[-0.051,-0.041]	[0.003,0.006]	[0.008,0.015]	[0.011,0.016]	[0.008,0.01]
MA: Add	-0.022**	-0.021**	0.142**	0.030**	-0.123**	-0.027**	0.021**
Comorbidities	[-0.024,-0.020]	[-0.023,-0.020]	[0.136,0.147]	[0.028,0.033]	[-0.127,-0.119]	[-0.031,-0.023]	[0.019,0.023]
MA: Complete	0.012**	-0.005**	-0.051**	0.006**	0.013**	0.015**	0.011**
Contracts Sample	[0.010,0.014]	[-0.006,-0.004]	[-0.057,-0.045]	[0.004,0.008]	[0.008,0.017]	[0.012,0.018]	[0.010,0.013]
Hospital Fixed	0.010**	-0.000	-0.052**	0.003**	0.016**	0.014**	0.010**
Effects	[0.009,0.012]	[-0.001,0.001]	[-0.055,-0.049]	[0.002,0.004]	[0.013,0.019]	[0.012,0.016]	[0.009,0.012]

Sample sizes for alternative comparison groups: main sample: 995212, intent to treat (includes switchers, MA status assigned 6 months prior to death): 1,024,730, complete contracts: 874155, hospital fixed effects: 858820.

eTable 11: Regression-Adjusted Differences in Post-Discharge Care Among Medicare Advantage versus Traditional Medicare Enrollees Hospitalized in the Last 6 Months of Life and Discharged Home, 2016 – 2018, Alternative Samples and Specifications.

	(1)<br>Home Health <= 3 Days Post-Discharge	(2)<br>Home Health <= 7 Days Post-Discharge	(3)<br>Home Hospice <= 3 Days Post-Discharge	(4)<br>Home Hospice <= 7 Days Post-Discharge
MA	0.042**	0.055**	0.013**	0.015**
	[0.038,0.046]	[0.051,0.059]	[0.008,0.017]	[0.010,0.020]
MA: Intent to	0.050**	0.065**	0.028**	0.031**
Treat	[0.046,0.054]	[0.061,0.068]	[0.023,0.032]	[0.027,0.035]
MA: Add	0.040**	0.054**	0.011**	0.013**
Comorbidities	[0.036,0.044]	[0.050,0.058]	[0.007,0.016]	[0.008,0.017]
MA: Complete	0.048**	0.063**	0.014**	0.017**
Contracts Sample	[0.044,0.052]	[0.058,0.067]	[0.011,0.018]	[0.013,0.020]
Hospital Fixed	0.041**	0.055**	0.010**	0.012**
Effects	[0.038,0.044]	[0.052,0.058]	[0.007,0.013]	[0.010,0.015]

Sample sizes for alternative comparison groups: main sample: 488555, intent to treat (includes switchers, MA status assigned 6 months prior to death): 603823, complete contracts: 474814, hospital fixed effects: 451219.

eTable 12: Regression-Adjusted Differences in Post-Discharge Care Among Medicare Advantage versus Traditional Medicare Enrollees Hospitalized in the Last 6 Months of Life and Discharged Home, 2016 – 2018, Alternative Samples and Specifications, Life-Limiting Conditions Sample

	(1)<br>Home Health <= 3 Days Post-Discharge	(2)<br>Home Health <= 7 Days Post-Discharge	(3)<br>Home Hospice <= 3 Days Post-Discharge	(4)<br>Home Hospice <= 7 Days Post-Discharge
MA	0.041**	0.055**	0.011**	0.013**
	[0.037,0.045]	[0.050,0.059]	[0.007,0.015]	[0.009,0.017]
MA: Intent to	0.049**	0.064**	0.026**	0.030**
Treat	[0.045,0.053]	[0.056,0.066]	[0.022,0.029]	[0.025,0.033]
MA: Add	0.039**	0.052**	0.009**	0.010**
Comorbidities	[0.035,0.043]	[0.048,0.057]	[0.005,0.013]	[0.007,0.014]
MA: Complete	0.046**	0.061**	0.014**	0.016**
Contracts Sample	[0.042,0.050]	[0.056,0.066]	[0.010,0.017]	[0.012,0.020]
Hospital Fixed	0.041**	0.054**	0.010**	0.012**
Effects	[0.038,0.044]	[0.051,0.058]	[0.007,0.013]	[0.009,0.015]

Sample sizes for alternative comparison groups: main sample: 398926, intent to treat (includes switchers, MA status assigned 6 months prior to death): 490291, complete contracts: 388265, hospital fixed effects: 368559.
