# Supplementary material for: Medicare-Covered Services Near the End of Life in Medicare Advantage vs Traditional Medicare
Source: JAMA Health Forum. 2024 Jul 19;5(7):e241777. doi: 10.1001/jamahealthforum.2024.1777 (PMC11259900; doi:10.1001/jamahealthforum.2024.1777)
Supplement: Supplement 2. — Data Sharing Statement [file jamahealthforum-e241777-s002.pdf]

Data Sharing Statement

Nicholas. Medicare-Covered Services Near the End of Life in Medicare Advantage vs Traditional Medicare. *JAMA Health Forum*. Published July 12, 2024. doi:10.1001/jamahealthforum.2024.1777

Data
Data available: No

Additional Information
Explanation for why data not available: The Centers for Medicare and Medicaid Services does not allow data sharing.
